# Left atrial function in obese and non-obese patients undergoing percutaneous pulmonary vein isolation

**DOI:** 10.1007/s00380-018-1243-0

**Published:** 2018-08-24

**Authors:** Małgorzata Cichoń, Joanna Wieczorek, Maciej Wybraniec, Iwona Woźniak-Skowerska, Andrzej Hoffmann, Seweryn Nowak, Krzysztof Szydło, Anna Wnuk-Wojnar, Katarzyna Mizia-Stec

**Affiliations:** First Department of Cardiology, Medical University of Silesia, ul. Ziołowa 45/47, Katowice, 40-635 Poland

**Keywords:** Obesity, Atrial fibrillation, Pulmonary vein isolation, Left atrial strain

## Abstract

Obesity constitutes a risk factor for atrial fibrillation (AF) and modifies the efficacy of invasive AF treatment. Left atrial (LA) global longitudinal strain (GLS), which is measured using speckle-tracking echocardiography (STE), is one of the new methods that are helpful in evaluating the function of LA. The aim of the study was to evaluate LA function in obese and non-obese patients that were undergoing percutaneous pulmonary vein isolation (PVI) before and 6 months after the procedure. 89 patients (F/M: 31/58; mean age: 55.8 ± 9.8 years) with paroxysmal or persistent symptomatic AF that had been qualified for percutaneous PVI were prospectively enrolled in the study. Body mass index (BMI) constituted as a discriminating factor for the study groups: obese group: BMI ≥ 30 kg/m^2^ (29 patients, F/M: 13/16, mean age: 55.13 ± 10.1 years) and non-obese group BMI < 30 kg/m^2^ (60 patients, F/M: 18/42, mean age: 57.17 ± 9.0 years). Transthoracic echocardiography (TTE) with LA GLS and segmental longitudinal strain were analysed 1 day before and 6 months after PVI. PVI efficacy was evaluated 6 months after PVI via a seven-day Holter monitoring. Baseline analysis revealed significantly lower two-chamber (2-Ch) LA GLS in the obese patients compared to the non-obese subjects (− 10.55 ± 3.7 vs − 13.11 ± 5.1, *p* = 0.004). Segmental strain analysis showed no significant differences between the groups. The data that was obtained 6 months after PVI showed a significantly lower 4-Ch LA GLS in the obese patients compared to the non-obese subjects (− 11.04 ± 5.0 vs − 13.91 ± 4.2, *p* = 0.02), which was accompanied by a significantly lower segmental 4-Ch LA function in the obese patients (med-sept: − 11.66 ± 11.2 vs − 15.97 ± 5.3, *p* = 0.04; api-sept: − 9.04 ± 6.3 vs − 13.62 ± 6.5, *p* < 0.001; api-lat: − 7.62 ± 4.0 vs − 13.62 ± 6.5, *p* < 0.001; med-lat: −9.31 + − 7.9 vs − 15.04 + −  6.3, *p* = 0.003, global: − 11.04 + − 5.0 vs − 13.91 + − 4.2, *p* = 0.02). PVI efficacy was confirmed in 52 (58.4%) patients and was similar in both groups. Comparison of the baseline and 6-month strain revealed no differences in LA GLS in either group. Differences in LA GLS before and after the procedure (delta LA GLS) were not obesity dependent. Apical-septal and apical-lateral strain in the obese group, which were measured in 4-Ch view, were significantly lower after the procedure compared to the baseline (*p* < 0.001). Obese patients with paroxysmal AF were characterised by impaired LA GLS, which is persistent and was accompanied by segmental dysfunction after PVI at the 6-month follow-up. PVI efficacy was comparable between the obese and non-obese patients.

## Introduction

Over two billion people worldwide are overweight or obese despite public policies and growing individual health-consciousness, which creates important health repercussions in addition to high treatment-related costs. Obesity is one of the most common causes of structural and functional cardiac remodelling that leads to its dysfunction. According to previous studies, obesity is a confirmed risk factor of many cardiovascular diseases including atrial fibrillation (AF). On the other hand, AF is the most common sustained arrhythmia in clinical practice and is a major cause of morbidity due to the associated risk of stroke.

Evaluation of left atrial (LA) function plays an important role in patients with AF. LA GLS and strain rate, which are measured using pulse wave Doppler echocardiography, are new non-invasive methods that are helpful in evaluating heart mechanics.

LA strain is the parameter independent from the LA dimension estimating the changes in myocardial fibres length. Deformation of LA in presented as a percentage of the output parameters. Using tissue Doppler and speckle-tracking echocardiography it is possible to asses not only global LA function but also estimate segmental function separately. Global strain as a resultant of segmental measurements is dependent on direction of the cumulative deformation.

Furthermore, strain rate imaging can identify LA dysfunction even before LA dilation or functional impairment as assessed by conventional tissue Doppler imaging [1]. There are some data that the assessment of LA deformation by strain rate imaging may be a predictor of the maintenance of a sinus rhythm after either electrical cardioversion or catheter ablation [2].

Despite a number of limitations regarding its clinical application, including the dependence of altered left ventricular hemodynamics, image quality, single plane assessment and the tethering effect, speckle-tracking echocardiography has been used as a non-invasive technique for assessing LA function in many cardiovascular diseases [3].

Taking into account the increasing number of obese patients with AF, as well as considering the optimal method for invasive procedures, the aim of the study was to evaluate LA function in obese and non-obese patients that were undergoing percutaneous pulmonary vein isolation (PVI) before and 6 months after the procedure.

## Methods

89 patients (males: 58/65%; mean age: 56 ± 9) with paroxysmal or persistent symptomatic AF that were hospitalised in the Department of Cardiology between 2014 and 2016 and who had qualified for percutaneous pulmonary vein isolation (PVI) were prospectively enrolled in the study. The main inclusion criterion for the study was a maintained sinus rhythm before pulmonary vein isolation and a preserved left ventricular ejection fraction. The primary endpoint of the study was the detection of any form of AF using Holter monitoring at the six-month follow-up. Patients with a history of transient ischemic attack or stroke of any kind, active neoplastic disease, inflammatory disease within past 3 months, chronic kidney disease with estimated glomerular filtration rate < 30 ml/min/1.73 m^2^, liver dysfunction (any hepatic aminotransferase > 3 × upper reference limit), valvular heart disease (moderate or severe), left ventricle ejection fraction < 50%, congestive heart failure and idiopathic cardiomyopathy were excluded from the study.

The patients were divided into two groups based on their body mass index. Body mass index, which constituted a discriminating factor for the study groups, was > 30 kg/m^2^ in the obesity group (OG) and < 30 kg/m^2^ in the non-obese group (NOG).

Transthoracic echocardiography (TTE) with left atrium global (LA GLS) and segmental wall peak longitudinal strains was performed 1 day before and 6 months after PVI. Measurements were performed during the left atrium contractile period and were based on the five-wall model for left atrium segmentation corresponding with a six-segment division in apical two- and four-chamber projections.

The study has been approved by the ethics committee and has been performed in accordance with the ethical standards laid down in the 1964 Declaration of Helsinki. All persons gave their informed consent prior to their inclusion in the study.

### Laboratory testing

On admission, all of the patients that were qualified for percutaneous pulmonary vein isolation underwent basic laboratory tests including morphology, electrolyte levels, kidney and liver function parameters, concentrations of lipids and glucose and inflammation markers. Blood samples (10 ml) were collected from the antecubital vein.

### Holter electrocardiomonitoring

A prolonged 24-h electrocardiomonitoring was performed prior to the procedure and 6 months after PVI using a Pathfinder SL system (Spacelabs Healthcare, Deerfield, WI, USA). PVI efficiency was assessed 6 months after PVI in a seven-day Holter monitoring and was defined as the absence of any form of AF.

### Echocardiography

Transthoracic echocardiography was performed 1 day before and 6 months after the procedure by a single experienced investigator using an Epiq 7G (Philips, Andover, MA, USA) with a 2.5-MHz probe in 2D, M, and Doppler modes. The routine check-up consisted of measurements of the heart chamber dimensions, the assessment of valvular function and myocardial contractility.

Transoesophageal echocardiography was performed in all of patient 24 h before the procedure to exclude the potential presence of an LA appendage thrombus.

Speckle-tracking imaging with LA strain analysis was performed using EchoPAC Software version 112.0.0 (GE Healthcare, Horten, Norway). The five-wall model for left atrium segmentation corresponding with a six-segment division in apical two- (2Ch) and four-chamber (4Ch) projections was used during the LA strain analysis [4].

### Definitions

AF was defined as arrhythmic episode showing typical pattern of irregular RR intervals and no discernible, distinct P waves lasting at least 30 s [5]. Paroxysmal AF was defined as self-terminating within 48 h or cardioverted within 7 days from the onset of arrhythmia. Persistent AF was defined as arrhythmia lasting for more than 7 days.

Obesity was defined as a body mass index equal to or higher than 30 kg/m^2^. Anthropological measurements were taken by medical personnel on the day of admission.

BSA was obtained using Mosteller formula.

Arterial hypertension was defined as newly recognised hypertension based on two separate measurements during hospitalisation that exceeded 140/90 mmHg, a previous hypertension diagnosis or any antihypertensive drug use [6].

Diabetes mellitus (DM) was diagnosed if the fasting blood glucose exceeded 125 md/dl in two separate measurements or in the case of the use of hypoglycemic agents. Impaired fasting glucose (fasting glucose over 100 md/dl) and impaired glucose tolerance (2-h glucose levels in 75 g-oral glucose test higher than 140 mg/dl) were analysed jointly with DM [7].

Kidney function was represented by estimated glomerular filtration rate (eGFR). eGFR was calculated according to the Cockroft–Gault formula including the creatinine level, patient age, sex and weight.

### Pulmonary vein isolation procedure

Every patient that was qualified for the procedure was administered vitamin K agonist anticoagulation therapy for at least 3 months before PVI. Protrombine time, which was represented as International Normalized ratio (INR), was assessed systematically to provide adequate anticoagulation. PVI was performed in patients with INR on the day of procedure up to 2.5 (preferably 2.0–2.5). All of the patients underwent trans-oesophageal echocardiography within 24 h before procedure to exclude the potential presence of an LA appendage thrombus.

Vein access that was necessary to perform the procedure was obtained using Seldinger technique. A quadripolar electrode placed in the right ventricle was induced through the left femoral vein, circular mapping and radiofrequency ablation electrodes or cryoablation balloon by the right femoral vein. Coronary sinus catheterisation was performed from the right jugular access.

At the beginning of the PVI, a left atrium rotational angiography was performed. To represent left atrium a contrast medium was injected into the pulmonary veins during fast right ventricular stimulation. A trans-septal puncture was performed under the control of fluoroscopy. The CARTO^®^3 system (Biosense Webster, Diamond Bar, CA, USA) was used to create a three-dimensional electro-anatomical mapping of left atrium. Under the guidance of a circular mapping electrode Lasso (Biosense Webster, Diamond Bar, CA, USA) or Achieve (Medtronic, MN, USA), seventy-seven patients underwent radiofrequency ablation using a ThermoCool^®^ SmartTouch^®^ SF catheter (Biosense Webster, Diamond Bar, CA, USA) and eleven individuals had balloon cryoablation using an Arctic Front Advance™ catheter (Medtronic, MN, USA).

Anticoagulation during the procedure included an intravenous bolus of unfractioned heparin, which was administered immediately after the trans-septal puncture and as a continuous infusion to obtain activation clotting time over 300 ms, which was verified every 30 min.

After the procedure, the 24-h heparin infusion was continued in patients with a non-therapeutic INR during the procedure. An oral anticoagulant was restored the same day, 4 h after the procedure to obtain adequate anticoagulation for at least 2 months after PVI.

### Statistical analysis

Statistica 10.0 (StatSoft Poland) software was used to perform the statistical analysis of the collected data. All of the statistical tests were two-tailed. Variables were presented as the mean value ± standard deviation (SD) or median with boundaries of the 1st and 3rd quartile (quantitative variables) or as a number and percentage (qualitative variables).

After verifying the type of distribution in the Shapiro–Wilk test Student’s test for normally distributed variables or the Mann–Whitney test were performed in the case of a nonnormal distribution.

Baseline and follow-up strain comparison was based on the Wilcoxon test for paired samples and ANOVA (analysis of variance).

Statistically significant *p* value in the study was every *p* < 0.05.

## Results

### Demographic and clinical characteristics

89 patients with paroxysmal (*n* = 80; 89.9%) or persistent (*n* = 9; 10.1%) symptomatic AF that had qualified for percutaneous PVI were prospectively enrolled in the study (29 individuals in OG vs 60 in NOG). The mean BMI in the study group was 28.7 kg/m^2^. Patients qualified for PVI were highly symptomatic, and most of them were in EHRA III class. The group was characterised by a low risk of ischemic stroke (CHA2DS2-VASc mean = 1). The most common co-morbidities were systemic hypertension (59.4%), coronary artery disease (20.3%) and diabetes mellitus (17.4%).

PVI efficacy was confirmed in 52 (58.4%) of the patients (Table 1).

**Table 1:** Demographic and clinical characteristics of obese and non-obese group

Variable	Absolute count and percentage or		
	Median and 25–75 percentile or		
	Mean ± standard deviation		
	Obese group	Non-obese group	
	*N* = 29	*N* = 60	
	(BMI: 33.9 ± 2.8 kg/m^2^)	(BMI: 26.5 ± 2.2 kg/m^2^)	
CHA2DS2-VASc [pts]	2 (2; 3)	1 (1; 2)	< 0.05
EHRA score	3 (2; 3)	3 (2; 3)	NS
Holter monitoring			
Maximal HR [1/min]	96 (78; 109)	103 (84; 115)	NS
Mean HR [1/min]	67 (57; 75)	65 (57; 75)	NS
Minimal HR [1/min]	51 (44; 54)	50 (45; 55)	NS
Transthoracic echocardiography			
Left atrial antero-posterior diameter [mm]	41 (38; 44)	39 (36; 41)	< 0.05
Left atrial volume index [ml/m^2^]	29.85 ± 8.7	25.2 ± 10.4	NS
Left ventricular ejection fraction [%]	58.64 (55; 60)	58 (55; 60)	NS
*E*/*e*’	8.8 (7.7; 11.3)	8.5 (6.4; 10)	NS
Baseline laboratory tests			
Platelet count [× 1000/mm^3^]	197 (164; 221)	195 (164; 217)	NS
Hemoglobin concentration [g/dl]	13.9 (12.5; 15.1)	14.3 (13.5; 14.9)	NS
White blood cells [× 1000/mm^3^]	5.86 (4.8; 6.9)	5.8 (4.9; 6.8)	NS
Thyroid-stimulating hormone [uIU/ml]	2.7 (1.6; 3.2)	2.5 (1; 2.7)	NS
Serum creatinine concentration [mg/dl]	0.89 (0.73; 1)	0.91 (0.83; 1.03)	NS
Estimated glomerular filtration rate [ml/min/1.73 m^2^]	82 (79; 90)	83 (75; 90)	NS
HsCRP [ng/dl]	2323 (987; 3012)	2389 (1002; 2881)	NS
Total cholesterol [mg/dl]	161 ± 37	186 ± 46	< 0.05

### Comparison between the obese and non-obese groups

The obese and non-obese groups differed significantly not only in weight and BMI, but also in weight and BSA. Laboratory tests showed no differences in most of the parameters, but paradoxically patients in the non-obese group had higher total cholesterol and LDL-cholesterol levels than the obese group. Although both groups were highly symptomatic (EHRA III), the non-obese group had a lower risk of ischemic stoke compared to the obese group (OG: CHA2DS2VASc mean = 2 vs NOG: CHA2DS2VASc mean = 1) (Table 1).

PVI efficacy was confirmed in 52 (58.4%) patients and was similar in both groups (OG: 52.2% vs NOG: 60.0%).

### Echocardiographic measurements before PVI

Compared to the non-obese patients (Table 1) the obese patients had a significantly increased LA diameter (41.04 ± 4.2 vs 38.04 ± 4.2 mm, *p* = 0.03), LA maximal volume (90.85 ± 23.1 ml vs 76.38 ± 26.8, *p* = 0.04), LA P volume (64.05 ml ± 17.8 vs 50.39 ± 21.4, *p* = 0.02), LA minimal area (17.27 ± 4.0 vs 14.69 ± 5.0, *p* = 0.015) and LA maximal area (26.24 ± 3.8 vs 22.97 ± 6.1, *p* = 0.03).

Strain analysis revealed significantly lower 2-Ch LA GLS (− 10.55 ± 3.6 vs − 13.11 ± 5.0, *p* = 0.04) in obese group. Segmental strain analysis showed no significant differences between OG and NOG (Table 2).

**Table 2:** Obese and non-obese group strain comparison and PVI influence on left atrial strain

	Before PVI			6 months after PVI			Before vs 6-month after PVI	
	Obese	Non-obese	*p*	Obese	Non-obese	*p*	Obese	Non-obese
Strain in two-chamber view								
Basal-inferior	− 17.52 ± .4.1	− 17.72 ± 6.3	NS	− 13.14 ± 13.6	− 18.33 ± 6	NS	NS	NS
Medial-inferior	− 14.49 ± 3.7	− 14.60 ± 5.7	NS	− 14.70 ± 5.5	− 13.66 ± 6.3	NS	NS	NS
Apical-inferior	− 9.93 ± 4.4	− 12.26 ± 6.8	NS	− 9.22 ± 4.3	− 10.18 ± 5.8	NS	NS	NS
Apical-anterior	− 10.22 ± 4.5	− 12.29 ± 8.3	NS	− 8.58 ± 4.7	− 11.42 ± 4.4	**< 0.05**	NS	NS
Medial-anterior	− 10.87 ± 4.7	− 12.82 ± 9.7	NS	− 12.18 ± 5.5	− 13.48 ± 5.4	NS	NS	NS
Basal-anterior	− 15.13 ± 5.3	− 14.59 ± 11	NS	− 13.65 ± 9.6	− 15.06 ± 6.2	NS	NS	NS
Global	− 10.55 ± 3.5	− 13.11 ± 5	**< 0.05**	− 12.08 ± 3.8	− 12.47 ± 3.6	NS	NS	NS
Strain in four-chamber view								
Basal-septal	− 14.12 ± 3.8	− 15.38 ± 6.7	NS	− 14.03 ± 9.2	− 16.93 ± 4.8	NS	NS	NS
Medial-septal	− 13.75 ± 4.3	− 14.58 ± 5.9	NS	− 11.66 ± 11.2	− 15.97 ± 5,3	**< 0.05**	NS	NS
Apical-septal	− 12.45 ± 5.7	− 9.40 ± 17.9	NS	− 9.04 ± 6.2	− 14.67 ± 5.7	**< 0.05**	**< 0.05**	NS
Apical-lateral	− 12.14 ± 7.2	− 10.31 ± 7	NS	− 7.62 ± 3.9	− 13.62 ± 6.5	**< 0.05**	**< 0.05**	**< 0.05**
Medial-lateral	− 11.14 ± 64.9	− 13.07 ± 7	NS	− 9.31 ± 7.8	− 15.04 ± 6.2	**< 0.05**	NS	NS
Basal-lateral	− 12.95 ± 6.4	− 15.67 ± 9.4	NS	− 16.06 ± 7	− 17.01 ± 6.1	NS	NS	NS
Global	− 10.77 ± 3.7	− 11.98 ± 5	NS	− 11.04 ± 5	− 13.91 ± 4.2	**< 0.05**	NS	NS

Statistically significant *p* values marked in bold

### Echocardiographic measurements after PVI

The 6-month follow-up showed an increased LA P volume—left atrial volume measured at the beginning of the P wave in ECG (64.06 ± 20.4 ml vs 49.28 ± 20.0, *p* = 0.009) in the obesity group.

Data obtained 6 months after PVI showed a significantly lower 4-Ch LA GLS in the obese patients compared to the non-obese patients (− 11.04 ± 5.0 vs − 13.91 ± 4.2, *p* = 0.009) that corresponded with a significantly lower segmental 4-Ch LA function in the obese patients (med-sept: − 11.66 ± 11.2 vs − 15.97 ± 5.3, *p* = 0.04; api-sept: − 9.04 ± 6.3 vs − 13.62 ± 6.5, *p *< 0.001; api-lat: − 7.62 ± 4.0 vs − 13.62 ± 6.5, *p* < 0.001; med-lat: − 9.31 + − 7.9 vs − 15.04 + − 6.3, *p* = 0.003, global: − 11.04 + − 5.0 vs − 13.91 + − 4.2, *p* = 0.02).

Two-chamber strain analysis showed significant differences in the api-ant strain (− 8.85 ± 4.7 vs − 18.33 ± 6.0, *p* = 0.03) (Table 2).

### PVI influence on LA strain

Comparison of the baseline and 6-month strain revealed no differences in LA GLS in both groups. The apical-septal and apical-lateral strain in the obese group that were measured in the 4-Ch view were significantly lower after the procedure compared to the baseline (*p* < 0.001) (Table 2).

The apical-septal and apical-lateral strain in the obese group and the apical-lateral strain in the non-obese group measured in the four-chamber view were significantly different after the procedure compared to the baseline (Table 3).

**Table 3:** Delta strain analysis in obese and non-obese group

	Group		*p*
	Obese	Non-obese	
	Mean; SD (Median)	Mean; SD (Median)	
Delta strain in 2CH			
Basal-inferior	− 2.62 ± 15 (0.14)	− 2.96 ± 10 (− 2.41)	NS
Medial-inferior	− 4.24 ± 10 (3.67)	− 1.77 ± 8 (− 1.76)	NS
Apical-inferior	− 1.94 ± 7 (− 0.89)	− 0.76 ± 9 (− 1.29)	NS
Apical-anterior	− 1.14 ± 7 (− 0.25)	− 1.33 ± 10 (0.47)	NS
Medial-anterior	− 3.85 ± 8 (− 3.91)	− 3.05 ± 10 (− 1.44)	NS
Basal-anterior	− 3.95 ± 10 (− 4.05)	− 2.47 ± 12 (− 0.96)	NS
Global	1.03 ± 8 (0.4)	− 0.3 ± 9 (− 0.8)	NS
Delta strain in 4CH			
Basal-septal	− 5.65 ± 10 (− 3.59)	− 4.2 ± 11 (− 2.15)	NS
Medial-septal	− 4.96 ± 10 (− 4.16)	− 3.94 ± 10 (− 0.91)	NS
Apical-septal	− 0.25 ± 9 (4.23)	− 4.89 ± 8 (− 3.82)	**< 0.05**
Apical-lateral	1.24 ± 8 (4.9)	− 5 ± 10 (− 4.52)	**< 0.05**
Medial-lateral	− 0.94 ± 12 (1.13)	− 4.08 ± 11 (− 4.81)	NS
Basal-lateral	− 6.53 ± 11 (− 5.72)	− 3.27 ± 12 (− 3.07)	NS
Global	− 0.33 ± 10 (− 0.2)	0.21 ± 9 (1.5)	NS

Statistically significant *p* values marked in bold

However, LA GLS in both groups after PVI where comparable to the basal measurements (Table 3).

Delta-strain analysis revealed that the differences in global strain before and after procedure were not BMI-dependent (Table 4).

**Table 4:** Comparison of delta LA GLS in the study groups

Variable	Mann–Whitney *U* test by variable BMI > 30 kg/m^2^						
	Marked tests are significant at *p* < 0.05						
	Rank sum	Rank sum	*U*	*Z*	*p* value	*Z* adjusted	*p*
	Obese	Non-obese					
Delta-2CH LA GLS	949.50	1976.50	598.50	− 0.558	0.576	− 0.558	0.576
Delta-4CH LA GLS	1094.00	2066.00	635.00	0.558	0.577	0.558	0.577

## Discussion

The epidemic of obesity that is associated with most cardiovascular diseases has become one of the major problems of contemporary medicine. The interplay of adipocyte dysfunction, maladaptive immune and inflammatory responses, coexisting low-grade inflammation and insulin resistance increases the cardiovascular risk in obese patients [8]. The excessive inflammatory response and myocardial fibrosis that are observed in overweight patients are risk factors of the recurrence of arrhythmia after sinus rhythm restoration. Assessment of the left atrium function before those procedures can be used to predict ablation efficacy.

In our study, the average BMI for NOG was 26.5 kg/m^2^ compared to 33.9 kg/m^2^ in OG. More exaggerated difference in patients BMI could affect the results, however, up-stream therapy is the first line of AF management. Obesity not only increases the rate of recurrence of AF after PVI, but also is linked to higher complication rate during the procedure. Intensive weight reduction with adequate management of other cardiovascular risk factors could lead to fewer arrhythmic recurrences. Intensive lifestyle intervention is recommended method in AF treatment and the reason why the differences in patients BMI could not be highly exaggerated [5].

In the present study, we analysed the LA global longitudinal and segmental strain in patients with paroxysmal AF that were undergoing PVI. The point of our interest was to determine the influence of obesity on LA function before and six months after the procedure. We found impaired LA GLS in the obese individuals compared to the non-obese patients. Regardless of the global impairment, there were no differences in the baseline segmental LA function between the obese and non-obese subjects. The second main finding of the study was data on the segmental impairment that was observed in obese patients after the PVI procedure.

The clinical characteristics of our study groups revealed LA enlargement in the obese subjects compared to the non-obese subjects. These data are in agreement with well-recognized observations that obesity may be a cause of LA dilatation [9]. On the other hand, the enlargement of LA may be both a consequence and a substrate to develop and sustain arrhythmia [10].

In our study, the LA enlargement was accompanied by a significantly lower LA GLS in 4CH in the obese patients. Significant differences in LA function between the obese and non-obese patients that were found in the echocardiography were also observed in other studies [11, 12]. In a Japanese study, data on conventional and two-dimensional speckle-tracking echocardiography were obtained from 134 hypertensive patients. The group was divided into two subgroups according to body mass index. The study showed a statistically significant relationship between body weight and LA function. LA strain was lower in the obese group compared to the non-obese individuals [12].

The discussion should take into consideration data on the results of invasive treatment.

In our study group, PVI efficacy was confirmed in 52 (58.4%) patients and was only slightly lower in obese subjects regardless of LA dilatation or a higher CHA2DS2-Vasc score. These results should be interpreted with caution. In the literature, there are much data confirming a worse PVI efficacy in obese subjects [13–15].

In the study involving 2715 patients undergoing PVI, the five-year ablation freedom from arrhythmia was decreased in obese patients (with obesity defined as BMI of 35 or more) [16].

In the meta-analysis including eight studies, the clinical relevance of LA strain in predicting the recurrence of arrhythmia were analysed. Patients with an AF recurrence after PVI were characterised by a lower LA strain, which makes strain analysis a useful tool for identifying patients with a lower probability of being sinus rhythm maintainers. LA strain with QRS-analysis and P-analysis using different software package such as Echo-Pac, QLab, TomTec and VVI showed similar results.

Similarly, in the study by Montserrat et al. [17], the pre-procedural patient characteristics, as well as the LA dimensions and function were associated with the effectiveness of ablation. In patients who underwent their first PVI, the systemic hypertension and LA expansion index that was derived from a 3D Echo were independent predictors of the elimination of arrhythmia, but in a group of patients after re-ablation, only age was correlated with the effectiveness of PVI. Although LA size and function were associated with the efficacy of the first PVI, in the study group undergoing a repeated procedure, LA enlargement was correlated with the recurrence of arrhythmia only in younger patients.

According to Montserrat et al., LA GLS was significantly lower in patients who underwent a second PVI compared to the healthy volunteers and individuals after the first PVI. This suggests that recurrent PVI procedures may impair LA function [18].

The LA mechanical dyssynchrony that is measured in speckle-tracking echocardiography, and also LA deformation that is assessed by strain rate imaging are predictors of the PVI efficiency [19, 20].

In our study, we analysed patients undergoing their first ablation procedure. However, we also observed some form of the worsening of the segmental LA strain after PVI, especially in the obese subjects.

On the other hand, in the study of Kobayashi, Okura et al. [21], strain analysis showed a significant improvement in strain rate after PVI compared to the baseline data.

It should be noted that successful PVI may lead to reverse atrial remodelling. In the presented study, PVI had no significant impact on LA GLS in the obese and non-obese patients; however, we are aware that the limited number of patients may have had an adverse influence on the reliability of the study.

In a study that was designed to identify the predictors of atrial function improvement after PVI reverse, atrial remodelling was defined as a reduction of the left atrial volume (LAV) index > 10% from the baseline to the follow-up. Reverse atrial remodelling was observed in the entire population but was more frequent and more pronounced in patients after the successful ablation for AF. Patients with atrial function improvement showed a significant decrease in their NT-proBNP levels, as well as a better systolic and diastolic function. Patient mass index and the LAV-index were confirmed as independent predictors of reverse atrial remodelling [22].

Our study revealed that impaired left atrial strain parameters in obese subjects are not only persistent but even more exaggerated after PVI, especially the segmental impairment of LA function that was present after PVI in obese individuals. Data on segmental LA strain in obese and non-obese subjects undergoing PVI are limited.

A study involving 100 patients with AF who underwent catheter ablation suggested that the lateral total strain is the most useful parameter for predicting the recurrence of arrhythmia. In a 4-month follow-up, patients with a recurrence of AF had a lower LA global and lateral total strain and a larger maximum LA volume index. The lateral total strain and LA volume index were identified as independent predictors of the recurrence of AF. The most useful parameter for predicting sinus rhythm maintenance was the total lateral strain (LA-LS) [23].

When analysing the influence of PVI on LA function, we should be aware that catheter ablation restores sinus rhythm by the formation of a scar that isolates the misfiring electrical signals in the left atrium. Some studies have suggested that different ablation techniques have various effects on myocardial function. The wall motion was significantly more depressed in WACA compared to the other techniques, which may be caused by the RF applications of the lateral wall with a higher baseline motion. The results of the study suggest that placing the ablation scar in regions with a high baseline motion resulted in a greater depression of active function, while ablation of the posterior wall was less disruptive [24].

## Limitations

The lack of a control group, the limited number of enrolled patients, heterogeneous group including patients with paroxysmal and persistent AF and the disadvantages of the techniques used to assess atrial function were the most important limitations of our study.

Although groups with paroxysmal and persistent AF differ in LA dimensions and its function, which could have significant impact on the study results, according to guidelines, PVI is effective in restoring and maintaining sinus rhythm in patients with both paroxysmal, persistent, and probably long-standing persistent AF [5].

Although the low resolution, high amount of reverberation and other technique parameters were the main problem in assessing LA function, the introduction of the new generation of ultrasound equipment, establishing a high frame rate for the second harmonic imaging compensated for those limitations. However, there are other techniques, for example, the strain rate and left atrial sphericity index [25] that can be used to express left atrial dysfunction. The results of the studies are promising, but more evidence of their usefulness is needed.

## Conclusion

The study showed significantly lower strain parameters measured in TTE 4CH in patients with severe obesity. Obese patients with paroxysmal AF were characterised by impaired strain LA parameters that were persistent and even more exaggerated after PVI at the 6-month observation, which may be related to scar formation. The decreased atrial function after PVI in patients with BMI > 30 kg/m^2^ suggests a higher risk of the recurrence of arrhythmia in the obesity group.
